# The relationship between readiness to change pain-related exercise participation and perceived work ability: a cross-sectional study of factory workers

**DOI:** 10.1186/s12891-021-04642-6

**Published:** 2021-09-06

**Authors:** Paul Shawcross, Melinda Lyons, Victoria Filingeri

**Affiliations:** 1 Connect Health, Floor 2, The Light Box, Quorum Business Park, Benton Lane, Newcastle Upon Tyne, NE12 8EU England; 2 University of Derby, Kedleston Road, Derby, DE22 1GB England

**Keywords:** Pain, Work, Exercise, Lifestyle, Health

## Abstract

**Background:**

Healthy lifestyle behaviours are associated with protection against health disorders and pain. Exercise participation is one such behaviour, associated with improved outcomes in those experiencing pain. Musculoskeletal pain is highly prevalent in the workplace, particularly in factory workers and associated loss of work function is recognised as having a great impact on individuals, society and the economy. A worker’s ‘readiness to change pain behaviour’ is an important factor to consider in achieving a healthy lifestyle behaviour and potentially improved function. This study aimed to examine the relationship between a cohort of factory workers ‘readiness to change pain behaviour’ such as exercise and their ‘perceived work ability’.

**Methods:**

A cross-sectional study design was used to establish the relationship between ‘readiness to change pain behaviours’ and ‘perceived work ability’. The Multidimensional Pain Related Change Questionnaire 2 (MPRCQ2) was used to measure readiness to change various pain behaviours including exercise. The Work Ability Index (WAI) was used to assess ‘perceived work ability’. Seventy-five factory workers, aged over 18 (66 male, 9 female) were recruited using convenience sampling between September–November 2019. Correlation and multiple regression were used for statistical analysis.

**Results:**

Mean WAI, MPRCQ2 and MPRCQ2 exercise component were 41.89 (SD 5.28), 4.26 (SD 1.01) and 4.40 (SD 1.69). MPRCQ2 and MPRCQ2 exercise component were not significant predictors of WAI in factory workers (*F* (2, 72) = 2.17, *p* > 0.001). There was no significant relationship between MPRCQ2 and WAI (rs = .09, *p* > .05). However, there was a significant positive relationship between MPRCQ2 exercise component and WAI (rs = .23, *p* < .05).

**Conclusions:**

This study suggests that readiness to change pain-related exercise participation has a positive association with ‘perceived work ability’. Further research should explore the causal relationship and consider strength training as a specific type of exercise.

## Background

Obesity, physical inactivity, poor dietary habits and insufficient sleep are recognised as the key lifestyle risk factors that should be tackled by health professionals to improve health outcomes [1] For example, worldwide an estimated 1.6 million deaths per annum can be attributed to physical inactivity [2].

Pain is an important health outcome associated with a lack of healthy lifestyle behaviours [3, 4]. Particularly, engagement with physical activity and exercise, with physical inactivity associated with increased back pain and disability [5] and more physical activity associated with less back pain [6]. A seminal paper [4] linked back pain and disability with various lifestyle factors such as poor sleep, inactivity and sedentary behaviour coupled with fear-avoidance beliefs. The association of such lifestyle behaviours and pain is further explained using the common-sense model [7] where a person believes that their body is damaged or vulnerable because they were told this by health professionals or society and subsequently avoid activity and movement as a result. This is an understandable common-sense behaviour which may lead to inactivity [7].

A series of review papers were released by the Lancet journal in 2018 calling for a change in how we prevent and treat one of the most common pain areas, low back pain [8]. The main call for action was around changing the beliefs and behaviour of health professionals, patients and society to move away from a biological understanding of low back pain which creates fear and unhelpful behaviours, towards a holistic biopsychosocial understanding with person-centred care, focusing on self-management and healthy lifestyles [8–10]. Other recent research has suggested a change in current musculoskeletal healthcare practice to focus on identifying lifestyle and behavioural risk factors, challenge unhelpful thoughts and beliefs that lead to unhealthy behaviours and provide support with adopting healthy lifestyle behaviours [3, 4, 11].

The Stages of Change Model [12] presents 6 stages of readiness to change behaviour: pre-contemplation, contemplation, preparation, action, maintenance and relapse. Described as a continuous cycle of an individual’s state of readiness change. An individual’s readiness to change and adopt self-management strategies is associated with improved coping [13]. A recent systematic review reported moderate-quality evidence supporting the use of behaviour change techniques to enhance exercise adherence in people with musculoskeletal pain [14].

Workplace disability is on the increase in the UK with more than 7 million working-age disabled people [15]. Pain has been identified as the leading cause of disability [15]. Work ability is defined as the balance between an employee’s individual resources and their work demands which can be used as a predictor of future sickness absence and work function [16]. Pain is recognised as a threat to work ability and function [17–19]. Poor self-efficacy [20], kinesiophobia (fear of movement) [21] and maladaptive pain behaviours [22] are all thought to reduce work ability in workers experiencing pain. Pain behaviours being behaviours which are learned or conditioned from previous pain experiences or secondary learning from others. However, there are still gaps in knowledge relating to populations of interest. Such as factory workers who have a high prevalence of musculoskeletal absence reported in the manufacturing and production industry [23]. Research is now starting to focus on prediction models for future sickness absence due to musculoskeletal disorders [24]. This current study will add to this body of research by identifying potential behavioural risk factors that need to be included in such prediction models for factory workers.

This study will explore the relationship between pain behaviour and perceived work ability in factory workers by assessing for the presence of key pain behaviours and readiness to change these behaviours. Focus will be given to readiness to change exercise participation.

The aim of this study was to examine the statistical relationship between a cohort of factory workers ‘readiness to change pain behaviours’ and their ‘perceived work ability’. With particular focus on readiness to change ‘exercise participation’. The secondary aim was to compare this relationship in workers that were experiencing musculoskeletal pain and those that were not experiencing musculoskeletal pain.

The study hypotheses were:

- H1: ‘Readiness to change pain behaviours’ has a statistically significant positive relationship with ‘perceived work ability’ in factory workers.
- H2: ‘Readiness to change pain behaviour related to exercise participation’ has a statistically significant positive relationship with ‘perceived work ability’ in factory workers.

## Methods

### Study design, setting and sample size

A correlational design was used to assess the relationship between the variables of ‘readiness to change pain behaviour’ and ‘perceived work ability’ in a cohort of factory workers and establish if ‘readiness to change pain behaviour’ was associated with ‘perceived work ability’. This research used quantitative data to provide statistical analysis in the form of correlation and multiple regression. A cross-sectional study design was used for estimating the prevalence of readiness to change multiple pain behaviours, as the exercise component was considered independently. The study setting was a factory in the UK which manufactures home and personal care products. A sample size of 85 was targeted to achieve 80% power for a medium effect size based on Cohen’s values of 0.1, 0.3 and 0.5 for small, medium and large effect sizes associated with Pearson correlation values reported [25].

### Participants

The sample population consisted of 75 factory workers from a total of 98 workers in the factory. Convenience sampling was used to recruit participants between September–November 2019. Participants gave written consent for use of their data in group analysis. Participant characteristics were collected for population, but not individual analysis. No other personal information was collected to maintain anonymity.

The 75 participants (66 male, 9 female) modal age range was 55–64. Inclusion criteria were; aged over 18, employed within the factory for more than 3 months, English speaking. Exclusion criteria also included people registered as vulnerable. See Table 1 for participant characteristics.

**Table 1:** Description of the study population

Characteristic		Percentage (%)	N
Sex	Female	12	9
	Male	88	66
Age Range	18–24	9.3	7
	25–34	17.3	13
	35–44	25.3	19
	45–54	20	15
	55–64	26.7	20
	65+	1.3	1
Time worked in factory	3–12 months	13.3	10
	1–3 years	22.7	17
	3–5 years	22.7	17
	5–10 years	17.3	13
	10–20 years	14.7	11
	20+ years	9.3	7
Job role	Machine Operator	33.3	25
	Technical Operator	42.7	32
	Palletiser Operator	2.7	2
	Despatch Operator	5.3	4
	Manufacturing apprentice	2.7	2
	Planner	8	6
	Line Leader	5.3	4
Reported Pain	Pain in past 7 days	60	45
	No pain in past 7 days	40	30
	Pain in past 3 months	80	60
	No pain in past 3 months	20	15
Area of pain	Lower back	38.3	23
(past 3 months)	Other body areas	61.7	37
Other diagnosed health condition	Yes	46.7	35
	No	53.3	40

### Process and procedure

Invitation to take part in this research was displayed on a notice board and tables in a canteen area. Participants were able to sign up via their line manager or attend a session during their working day, where a researcher was present.

After an initial explanation from one of two researchers, reading of a participant information sheet and a chance to ask questions, the participant was left alone in a room for 20 min, to complete all questionnaires. The link to the Qualtrics questionnaire was distributed using a tablet computer.

Qualtrics guided the participants through the consent process and various inclusion/exclusion criteria. If participants did not meet the criteria or provide written consent, the questionnaire was stopped (*N* = 2). Participants that did meet inclusion criteria (*N* = 77) and then consented (*N* = 75) were taken through the WAI and MPRCQ2 questionnaires followed by a final study debrief. To attain external reliability, this same procedure was used with each participant.

The details and answers for all participants were stored securely by Qualtrics. This data was cleansed and exported to SPSS for statistical analysis. SPSS analysis was completed on a secure password-protected laptop computer.

### Exposure and comparisons

The WAI was used to measure ‘perceived work ability’. This questionnaire has high validity and reliability [26]. The WAI gives a score from 2 to 49 and four categories; ‘poor’ (2–27), ‘medium’ (28–36), ‘good’ (37–43), ‘very good’ (44–49), corresponding with 4 actions; ‘reinstate work ability’ (2–27), ‘improve work ability’ (28–36), ‘support work ability’ (37–43), ‘maintain work ability’ (44–49).

The MPRCQ2 was used to measure readiness to change various pain management behaviours. This questionnaire has good validity and reliability [27, 28]. The MPRCQ2 gives a score from 1 to 7 using 69 questions covering 9 behaviour components; use of relaxation, cognitive control, assertive communication, exercise participation, avoid resting due to pain, avoid regularly asking for assistance, task persistence, pacing and use of taught body mechanics. For the first of two sections, 1 represents *‘I am not doing this now, and am not interested in ever doing it.’* and 7 represents *‘I have been doing this for a long time (at least 6 months.)*’*.* The second section inverts the scores, so 1 represents *‘I am doing this now and am not interested in ever stopping’* and 7 represents ‘*I have not done this for a long time (at least 6 months)’.* An average score for each of the 9 behaviour components is calculated 1–7 and an overall score 9–63 [28]. The overall score and the ‘exercise participation’ behaviour component score were used in this study.

Participants reported if they were currently experiencing musculoskeletal pain or not, to allow for comparison between these two groups. Other participant characteristics measured included; sex, age range, shift pattern, job role, time in current job role, current or recent pain experience and other current health issues.

### Statistical analysis

Descriptive statistics were completed on ‘perceived work ability’ and ‘readiness to change pain behaviours’. Average scores with standard deviations and distribution of data were reported. A correlational design was chosen to assess the relationship between two variables, both ‘readiness to change pain behaviour’ with ‘perceived work ability’ and ‘readiness to change pain behaviour specific to exercise’ with ‘perceived work ability’. A correlational design was also used to assess the variables for 2 groups; participants reporting a pain experience in the past 7 days (*n* = 52) and those that had not reported a pain experience in the past 7 days (*n* = 23).

A multiple regression analysis was completed to test both ‘readiness to change pain behaviour’ and ‘readiness to change pain behaviour specific to exercise’ as predictors of ‘perceived work ability’. SPSS (IBM, Armonk, NY) was used for statistical analysis.

## Results

**Fig. 1:**
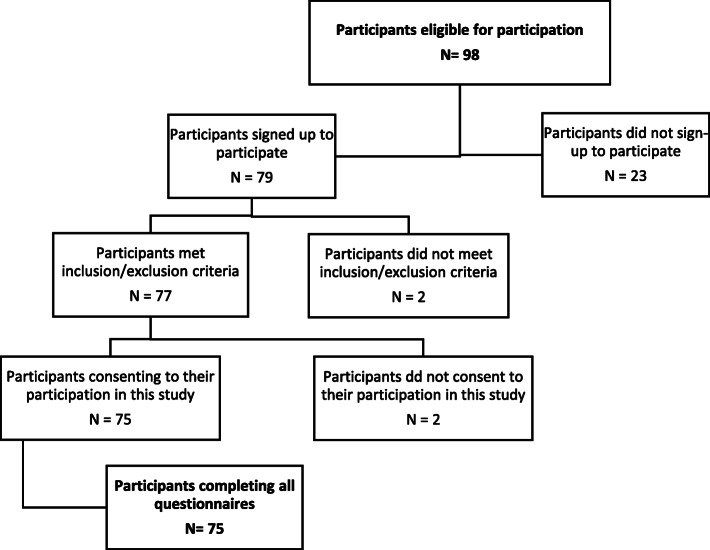
Flow chart of participant recruitment for this study

Two potential participants were excluded because they were employed within the factory for less than 3 months and 2 potential participants did not consent after reading the participant information sheet. Giving a total of 75 participants participated in this study (see Fig. 1). Descriptive details of the participants are presented in Table 1. Table 2 presents descriptive stats for WAI, MPRCQ2 and MPRCQ2 exercise component. Mean average WAI score was 41.89 (SD 5.28) which is categorised as a ‘good’ level of work ability [26] with a range of 28 to 49. Mean average MPRCQ2 score was 4.26 (SD 1.01) with a range of 2.1 to 6.6 and MPRCQ2 exercise component score was 4.40 (SD 1.69) with a range of 1 to 7.

**Table 2:** Descriptive statistics for WAI, MPRCQ2 and MPRCQ2 exercise component

	Mean (SD)	Min	Max	Z Skewness	Z Kurtosis	K-S Test	S-W Test	N
**WAI**	41.89 (5.28)	28.0	49.0	3.2	.66	.13	.93	75
**MPRCQ2**	4.26 (1.01)	2.1	6.6	.92	0.76	.08	.98	75
**MPRCQ2 - exercise**	4.40 (1.69)	1.0	7.0	.74	1.95	.09	.96	75

Skewness scores for WAI did not fall within the +/− 1.96 Z score range to meet assumptions of normality of data, Z > 3 (see Table 2). Non-parametric testing was used.

Correlations between the variables are shown in Table 3. The results showed that there was a positive relationship between MPRCQ2 and WAI, though this was not significant (rs = .09, *p* > .05). There was a significant positive relationship between MPRCQ2 exercise component and WAI (rs = .23, *p* < .05). This shows that ‘readiness to change pain behaviour specific to exercise’, has a positive association with ‘perceived work ability’.

**Table 3:** Correlation coefficients and significance levels for MPRCQ2 and MPRCQ2 exercise component independently against WAI for all participants, participants in pain and participants not in pain

	MPRCQ2	MPRCQ exercise component
**WAI – all participants**	.09 (.46)	.23 (.05)
**WAI – participants experiencing pain**	.12 (.41)	.18 (.19)
**WAI – participants not experiencing pain**	.10 (.65)	.41 (.052)

Tests to see if the data met the assumption of collinearity indicated that multicollinearity was not a concern (MPRCQ2 VIF = 1.49; MPRCQ2 exercise component, VIF = 1.49). The data met the assumption of independent errors (Durbin-Watson value = 1.89).

Data were analysed using a Multiple Regression using the Enter Method. The regression equation produced a small effect size (*R*^*2*^ = .06, *R*^*2*^_*Adj*_ = .03), indicating that ‘readiness to change pain behaviour’ (when all components are measured together) was not a significant predictor of ‘perceived work ability’ (*F* (2, 72) = 2.17, *p* > 0.001). ‘Readiness to change pain behaviour’ was not a significant predictor of ‘perceived work ability’ (*t* = .29, *df* = 74, *p* = 0.776). ‘readiness to change pain behaviour related to exercise participation’ participation was not a significant predictor of ‘perceived work ability’ (*t* = 1.53, *df* = 74, *p* = 0.131).

Data were split into 2 groups; participants reporting a pain experience in the past 7 days (*n* = 52) and those that hadn’t reported a pain experience in the past 7 days (*n* = 23). For participants in pain, correlations between the variables are shown in Table 3. The results showed that there was a positive relationship between MPRCQ2 and WAI, though this was not significant (rs = .12, *p* > .05). There was a positive relationship between MPRCQ2 exercise component and WAI, though this was not significant (rs = .18, *p* > .05). For participants not in pain, correlations between the variables are shown in Table 3. The results showed that there was a positive relationship between MPRCQ2 and WAI, though this was not significant (rs = .10, *p* > .05). There was a positive relationship between MPRCQ2 exercise participation and WAI, though this was not significant (rs = .41, *p* > .05).

## Discussion

This study hypothesised that ‘readiness to change pain behaviour’ has a statistically significant positive relationship with ‘perceived work ability’ in factory workers. The results indicated that this positive relationship was not statistically significant. Therefore, we accept the null hypothesis. This study hypothesised that ‘readiness to change pain behaviour related to exercise participation’ has a statistically significant positive relationship with ‘perceived work ability’ in factory workers. The results indicated that there was a statistically significant positive relationship. Therefore, this hypothesis was accepted. The secondary aim of this study was to compare the statistical relationship between factory workers ‘readiness to change pain behaviour’ and their ‘perceived work ability’ in workers that were experiencing musculoskeletal pain and those that were not experiencing musculoskeletal pain. The results reported no statistically significant relationship for either group.

A similarly unsupportive association was reported between readiness to self-manage pain and both physical and psychological functioning [13]. Although similar, the measure used was different, using the pain stages of change questionnaire (PSOCQ) instead of MPRCQ2 [13] which focuses on readiness to adopt a self-management approach rather than focusing on specific behavioural components like MPRCQ2. Patient disability and depression were the functional focus [13] opposed to ‘perceived work ability’ as in this current study. One study [29] used the MPRCQ2 to measure readiness to change and assess the association with pain-related function (rather than ‘perceived work ability’). No significant association was reported with the MPRCQ2 or any subcomponents (including exercise) with pain-related functioning. The cohort recruited [29] had some key differences with this current study populations as they all had low back pain and were general population rather than factory workers.

In contrast to this current study’s results data reported by Pitt-Catsouphes [30] in support of a statistically significant relationship between health behaviour changes influencing physical health and work ability (measured by WAI). Various influential review papers are supportive of behaviour change, exercise and self-management strategies for managing pain and improving function at work [4, 8–10, 31]. This body of research is specific to low back pain rather than musculoskeletal pain as a whole and of these 5 studies, only NICE [31] completed a robust systematic literature review. Behaviour change and ‘readiness to change pain behaviour’ may be more important for people experiencing low back pain than other musculoskeletal pain. Equally, this could be because incidence and consequently research is more prevalent in low back pain compared to other pain conditions. With 23 (38.3%) of 60 participants reporting that their pain included low back pain (see Table 1), this current study does not provide conclusive data. More primary research with large sample populations and subsequent systematic reviews are recommended focusing on musculoskeletal pain, other than low back pain.

The statistically significant positive relationship between ‘readiness to change pain behaviour related to exercise participation’ and ‘perceived work ability’ builds on the already established research base linking higher levels of physical activity and exercise as a key lifestyle behaviour change for improving pain and function associated with musculoskeletal pain [4, 6, 8–10, 14, 31]. ‘Readiness to change’ is a new area of focus and the specific relationship between ‘readiness to change pain behaviour related to exercise participation’ and ‘perceived work ability’ was not reported in these previous review papers, highlighting a new finding to add to the research base and investigate further. Applying these results practically, readiness to participate in exercise seems to be a positive behaviour and higher perceived work ability seems to be a positive perception. Both of which should be encouraged.

The mean average WAI score was categorised as a ‘good’ level of work ability [26] and seemed generally high for this cohort of factory workers, with no workers scoring in the lowest category. This mean average was similar to previous research mean average WAI scores of 42.2 (SD 4.2) [32] and 39.3 (SD 3) [33]. However, this current research presents a larger standard deviation for WAI scores, highlighting a greater spread of WAI scores. This may be related to this data not being normally distributed. Mean average MPRCQ2 in this study were similar to the original research populations mean average MPRCQ2 scores of 4.29 (SD 1.68). The MPRCQ2 exercise component was higher in this cohort of factory workers; 4.40 (SD 1.69) compared to the original cohort; 4.25 (SD 1.57) [28]. This difference may be the reason for the positive association with ‘perceived work ability’ reported in the results of this current study. Future research with different study populations is recommended to explore this.

No other research presents ‘readiness to change pain behaviour’ as a predictor of ‘perceived work ability’. Several studies have linked exercise participation (as a behaviour, but not readiness to change) with work ability. A systematic review and meta-analysis on workplace health promotion on wellbeing and work ability was completed in 2008 [34]. Exercise was reported to increase both wellbeing and work ability of workers. A similar systematic review conducted more recently [35] reported moderate-quality evidence for exercise and lifestyle education as a workplace intervention. Research considering behaviour change specific to exercise and workability was reported on [30] in support of a predictor relationship between ‘behaviour change related to exercise participation’ and ‘perceived work ability’. With changes in health behaviour specific to exercise associated with a statistically significant improvement in physical health and work ability [30]. ‘Readiness’ to change was not considered [30].

Exercise as a health behaviour has been considered as part of a paradigm for health performance [36], where exercise was 1 of 5 health behaviours considered to contribute to health performance. The authors [36] discuss attitudes as a key influencer of behaviour. They do not consider where readiness to change fits into this paradigm. The results from this current study suggest ‘readiness to change pain behaviour related to exercise participation’ should at least be considered. The exact nature of this association is still unclear and there does not appear to be a predictor relationship between ‘readiness to change pain behaviour’ and perceived work ability, based on the results of this current study.

The breakdown of the significant correlation between ‘readiness to change pain behaviour related to exercise participation’ and ‘perceived work ability’ into those with and without pain, is suggestive, although not statistically significant. Table 3 presents correlation coefficients and significance levels for participants experiencing pain .18(.19) and participants not experiencing pain .41(.052). Although not statistically significant, a difference between the two groups is presented here. The 23 participants not experiencing pain were likely too small in number to find the larger effect size (.41) as significant. The immediate inference would be that if a worker already has pain, they may be less ready participle in exercise as an effective behaviour for improving their pain and work ability. Whereas those who are not experiencing pain, are more likely to be ready to participate in exercise. Thus, exercise intervention programmes should target factory workers when they are not experiencing pain and are more likely to be ready to participate.

A previous study with less supportive findings was a randomised controlled trial (RCT) of 66 slaughterhouse workers with upper limb pain and work disability [33]. Workers had either 10 weeks of strength exercise or ergonomic training. Strength exercise was reported as superior for preventing deterioration of work ability but not improving work ability. This was an insignificant finding with low effect size, although this may be due to the small sample size. The researchers [33] also focused on strength training as a particular type of exercise in contrast to any exercise participation in this current study. One previous study did report conflicting results [37] using a subset of 80 participants from a larger cohort study [38] to compare engagement in positive lifestyle behaviours between 36 adults with chronic low back pain and 44 adults with no history of chronic low back pain. The health literacy measurement scale was used, and results present a greater difficulty engaging in positive lifestyle behaviours for those in chronic low back pain compared to those without. The authors [37] assessed the comparison between the two groups in which this current research does not. However, a small convenience sample was used [37], made up of people from the same, middle-class geographical area in Australia. Subsequently, population validity was low, and the sample may be subject to selection bias. Both of these previous studies [33, 37] assess exercise as an intervention rather than readiness to change. Further research is needed, comparing readiness to change lifestyle behaviours in people experiencing pain and people not experiencing pain.

### Limitations

A sample size of 85 was needed for 80% power for a medium effect size [25]. The sample of 75 participants used in this study gave reasonable power to find the small-medium correlation (0.23) significant between MPRCQ2 exercise component and work ability. The 23 participants not experiencing pain were too small in number to find the larger effect size (0.41) as significant. Convenience sampling was used due to the nature of the study design and the small study population available. Convenience sampling does expose the study findings to selection bias and reduces population validity. For example, the researchers were unable to recruit any workers that were currently absent from work. All participants were currently at work and so the results of this study can only be applied to workers in work, not those absent. The controlled workplace environment where participants completed the survey, with the researcher partially present may have unwittingly led to researcher bias which is a threat to internal validity. A lack of normality of WAI data was identified for this sample of workers and so non-parametric testing was used. Non-parametric testing is less powerful for detecting variability in data [39].

The MPRCQ2 was chosen for this study due to the focus on specific behavioural components like exercise. Yet, the 9 behaviour components may need an update. ‘Use of taught body mechanics’ [28] for example has come under scrutiny as a risk factor for musculoskeletal pain. This change in understanding was well summarised by a recent systematic review [40] reporting that lumbar spine flexion when lifting was not a risk factor for low back pain onset, persistence or a differentiator for people in pain. Other important risk factors for pain, such as diet [36], sleep [11], smoking [10, 11] and obesity [10, 11] have also emerged but are not considered by MPRCQ2. This could be considered a limitation of the MPRCQ2 and subsequently this study. Future development and inclusion of these risk factors are recommended for any tool measuring ‘readiness to change pain behaviour’.

### Directions for further research

The concept of readiness to change was first discussed in 1983 [12], yet it has not been studied in great depth. Its importance was highlighted for adopting self-management strategies for managing chronic pain [13]. In contrast, Byrka & Kaiser [36] discuss health attitudes as a key influencer of health behaviour rather than readiness to change. Consensus is yet to be agreed on which individual characteristics (readiness, attitudes, motivation, beliefs and expectations) are most important for health and pain behaviour change. The results from this current study suggest ‘readiness to change pain behaviour related to exercise participation’ should at least be considered although the exact nature of this association is still unclear. The range of potential behaviour influencing characteristics may be the reason that readiness to change has not been studied in great depth since it was first proposed [12]. All are likely to play some role and further research is needed to clarify how these characteristics interact. Further comparison of ‘readiness to change pain behaviour related to exercise participation’ in workers experiencing pain and workers not experiencing pain is recommended to explore if the larger effect size identified for those not in pain is statistically significant for a larger sample size.

The results of this current study add weight to the current evidence base which already recognises exercise as a positive behaviour for improving pain and function associated with musculoskeletal pain [4, 6, 8–10, 14, 31]. It may be useful for future research to investigate which specific type of exercise is most beneficial for workers for improving pain and work ability. Early research in this area is suggestive of strength training emerging as the most effective exercise for managing musculoskeletal pain amongst workers [41–44]. None of the papers included in these systematic reviews [41–44] considered work ability and this is recommended as a sensible focus for future research [43].

A prediction model was developed [24], for future sick leave and loss of work function due to musculoskeletal pain. Using the occupational health check questionnaire to measure predictor variables such as psychological distress, work pace and presence of musculoskeletal complaints, but this questionnaire does not consider exercise, lifestyle behaviours or readiness to change. These variables are subsequently missing from the prediction model. The findings of this current study would suggest that these two research areas would benefit from coming together; readiness to change exercise behaviour and work ability of workers with musculoskeletal pain.

## Conclusions

This research does not provide support for an association or predictor relationship between factory workers ‘readiness to change pain behaviour’ and their ‘perceived work ability’. This research does provide support for a positive relationship between factory workers ‘readiness to change pain behaviour related to exercise participation’ and their ‘perceived work ability’. In a practical sense, readiness to participate in exercise seems to be a positive behaviour and may be constructive for improving ‘perceived work ability’. Equally, the higher perceived work ability seems to be a positive perception and may impact on readiness to participate in exercise. Both behaviours and perceptions should be encouraged and supported in a clinical setting.

This research did highlight a larger effect size for ‘readiness to change pain behaviour related to exercise participation’ in workers not experiencing pain compared to workers experiencing pain. Although not statistically significant, this is suggestive that workers not experiencing pain may have higher readiness to change pain behaviour related to exercise participation and should be targeted with proactive intervention. This is an area for further research using a larger sample size.

Although this finding doesn’t represent causation, this gives a good starting point for further research. To enhance the internal validity and population validity of these findings, future research should apply a methodology which reduces use the potential for selection and researcher bias and look to recruit a larger and more varied sample.

Further research should explore the causal relationship between readiness to change exercise participation as a pain behaviour and ‘perceived work ability’. This research should consider motivations, attitudes, beliefs and expectations in relation to exercise participation as well as readiness to change. Workers that are absent from work should also be included to compare differences. Given the emerging evidence base for strength training for rehabilitating workers with musculoskeletal pain, this specific type of exercise should be applied to future research exploring an association with work ability.

## Data Availability

The raw data and datasets used and/or analysed during the current study are available from the corresponding authors upon reasonable request.

## Abbreviations

MPRCQ2: Multidimensional Pain Related Change Questionnaire 2
WAI: Work Ability Index
PSOCQ: Pain stages of change questionnaire
NICE: National Institute for Health and Care Excellence
